# Experiences of sexuality of older cisgender gay men: a systematic review and meta-synthesis of qualitative studies

**DOI:** 10.1590/0102-311XEN228324

**Published:** 2025-07-25

**Authors:** Diogo Sussumu Okasawara, Letícia Carolina Boffi, Érika Arantes Oliveira-Cardoso, Manoel Antônio dos Santos

**Affiliations:** 1 Faculdade de Filosofia, Ciências e Letras de Ribeirão Preto, Universidade de São Paulo, Ribeirão Preto, Brasil. Faculdade de Filosofia, Ciências e Letras de Ribeirão Preto Universidade de São Paulo Ribeirão Preto Brazil

**Keywords:** Men’s Health, Aged, Sexuality, Homosexuality, Systematic Review, Saúde do Homem, Idoso, Sexualidade, Homossexualidade, Revisão Sistemática, Salud del Hombre, Anciano, Sexualidad, Homosexualidad, Revisión Sistemática

## Abstract

This paper presents a systematic review and meta-synthesis to synthesize and reinterpret findings of primary qualitative studies on older cisgender gay men’s experience of sexuality and seeking healthcare services. Following the SPIDER search strategy and PRISMA guidelines, bibliographic search was conducted on the CINAHL, LILACS, Oasisbr, APA PsycInfo, PubMed/MEDLINE, Scopus and Web of Science databases. Two independent researchers screened and selected the articles, collected data and elaborated a thematic synthesis. Of the 2,382 studies identified in the databases, 30 articles met the inclusion and exclusion criteria and were selected, totaling 849 gay men between 50 and 95 years old. Analysis produced four descriptive themes: (1) Living history: perceptions on the impact of historical and cultural development on experiencing sexuality; (2) Aging as a gay man: understandings on becoming and being a new body and self; (3) Caring for a social network: severing, building and maintaining bonds; (4) Handling health and professional care. From it were developed three analytical themes: (1) (Un)doing gay men through a dehumanizing gaze; (2) Oldness as performance; (3) To care is to care about. The discriminatory manner society perceives older gay men produces specific isolation from public life, gay communities, and health care services. Punitive laws against their sexuality, negative media representation, lack of proper care and family, community, and spiritual support interfered with their self-care and care seeking habits. Culturally sensitive future measures in policymaking and healthcare services are needed to properly care for this population.

## Resumo

Este artigo buscou apresentar uma revisão sistemática e metassíntese para sintetizar e reinterpretar os achados de estudos qualitativos primários sobre a experiência da sexualidade e a procura por serviços de saúde entre idosos gays cisgêneros. A revisão sistemática da literatura foi realizada com base na estratégia de busca SPIDER e nas diretrizes PRISMA em sete bases de dados: CINAHL, LILACS, Oasisbr, APA PsycInfo, PubMed/MEDLINE, Scopus e Web of Science. No todo, dois pesquisadores independentes selecionaram e avaliaram os artigos, coletaram dados e elaboraram uma síntese temática. Um total de 2.382 estudos foram recuperados dos bancos de dados, dos quais 30 artigos envolvendo 849 homens gays com idade entre 50 e 95 anos que atenderam aos critérios de inclusão/exclusão foram selecionados. Foram produzidos quatro temas descritivos: (1) História viva: percepções sobre o impacto do desenvolvimento histórico e cultural na vivência da sexualidade; (2) Envelhecer como homem gay: compreensões sobre tornar-se e ser um novo corpo e eu; (3) Cuidar de uma rede social: cortar, construir e manter vínculos; (4) Lidar com cuidados de saúde e profissionais. No todo, três temas analíticos foram desenvolvidos: (1) (Des)fazer gays através de um olhar desumanizador; (2) Velhice como performativa; (3) Cuidar é se importar. A forma discriminatória com que os gays idosos são percebidos pela sociedade os isola da vida pública, comunidades gays e serviços de saúde. Leis punitivas à sua sexualidade, representação negativa na mídia e falta de cuidados adequados e apoio familiar, comunitário e espiritual interferiram em seus hábitos de autocuidado e busca de cuidado. Assim, medidas futuras culturalmente sensíveis na formulação de políticas e serviços de saúde são necessárias para cuidar adequadamente dessa população.

## Resumen

El objetivo de este artículo es presentar una revisión sistemática y metasíntesis para recopilar y reinterpretar los hallazgos de estudios cualitativos primarios sobre la experiencia de la sexualidad y la búsqueda de servicios de salud entre hombres mayores homosexuales cisgénero. La revisión sistemática de la literatura se condujo sobre la base de la estrategia de búsqueda SPIDER y las directrices PRISMA en siete bases de datos: CINAHL, LILACS, Oasisbr, APA PsycInfo, PubMed/MEDLINE, Scopus y Web of Science. Dos investigadores independientes examinaron y evaluaron los artículos, recopilaron los datos y elaboraron una síntesis temática. Se recuperaron un total de 2.382 estudios de las bases de datos. Un total de 30 artículos que cumplieron con los criterios de inclusión/exclusión fueron seleccionados, los cuales incluyeron 849 hombres homosexuales con edades comprendidas entre 50 y 95 años. Se elaboraron cuatro temas descriptivos: (1) Historia viva -percepciones sobre el impacto del desarrollo histórico y cultural en la experiencia de la sexualidad; (2) Envejecer como hombre gay -comprensiones sobre cómo transformarse y ser un nuevo cuerpo y un nuevo yo; (3) Cuidar de una red de relaciones sociales -cortar, construir y mantener vínculos; (4) El manejo de la atención sanitaria y profesional. Se desarrollaron tres temas analíticos: (1) (Des)hacer a los hombres homosexuales a través de una mirada deshumanizante; (2) La vejez como performativa; (3) Cuidar es preocuparse. La forma discriminatoria en que la sociedad percibe a los hombres homosexuales de edad avanzada produce un aislamiento específico de la vida pública, de las comunidades homosexuales y de los servicios de atención médica. Las leyes punitivas a su sexualidad, la representación negativa en los medios de comunicación, la falta de atención adecuada y de apoyo familiar, comunitario y espiritual interfirieron con sus hábitos de autocuidado y búsqueda de cuidado. Por lo tanto, se requieren medidas futuras culturalmente sensibles en la formulación de políticas y en los servicios de atención médica para atender adecuadamente a esta población.

## Introduction

According to the World Health Organization (WHO), sexuality is a complex and multi-layered phenomenon that encompasses sexual activities, reproduction, gender identities and roles, sexual orientation, the domains of pleasure and intimacy, and is affected by the interaction between psychological, biological, economic, political, legal, cultural, social, historical, religious and spiritual factors ^1^. The growing debate around sexual health ^2^ has placed sexuality as a crucial sphere for establishing health and human rights. It highlights the idiosyncrasies of sexuality within each group based on their geographic and cultural setting, and focus on the particular health needs and issues which impact well-being that may emerge. Aligned with a definition of health ^3^ that goes beyond the absence of disease or infirmity to include a physical, mental and social well-being state, research on sexual health can produce knowledge to support the development of effective health care schemes. The notion of sexual justice ^4^ enriches this perspective by emphasizing the social inequalities it entails, calling for research action dedicated to actively resist against oppressive conditions, interfering on power dynamics by reporting these conditions and producing and providing access to proper care strategies.

Given the complex nature of research topics on sexual health and its proximity to the political adversities minorities face, the perspective of intersectionality can act as a guideline to developing and conducting research in this area ^5^. Intersectionality has been widely used as an approach to critically evaluate knowledge and praxis about minorities, especially regarding questions related to class, gender, and race ^6^. Recently, intersectionality grew as a field and also encompasses sexuality, religion, nationality, ethnicity and age ^6^.

When it comes to gay men’s sexual health, investigations have extensively and continuously been made on HIV ^7^. Despite its importance, this tendency led health professionals to overlook other possible needs beyond sexually transmitted infections (STI) management when caring for gay men ^8^. Regarding the sexuality of older adults, researchers have elected them as group to be focused on when it comes to sexual health given how they’re usually perceived as sexually inactive and have their specific needs neglected ^9^. Considering that society is bridging towards a future in which population is majorly composed by older people ^10^, the relevance and impact of knowledge about their sexual health tends to increase. Studies on the intersection between gay men and older adults is a recent, expanding research field ^11^. Although literature review studies were conducted on the sexual expression of older adults, they do not focus on older cisgender gay men or their perspective ^12^^,^^13^. Studies about LGBTQIAPN+ and cisgender men’s sexual health indicate that research on subpopulations within these groups remains underdeveloped ^14^^,^^15^. Lack of systematized knowledge on this topic hinders the development of public health policies intended to improve resources and protective factors.

Thus, this paper presents a systematic review and meta-synthesis that summarized and reinterpreted findings of primary qualitative studies on the sexuality of older cisgender gay men.

## Method

### Design

The systematic review and meta-synthesis were conducted following ten steps ^16^^,^^17^: (1) development of the research question using the SPIDER strategy; (2) definition of selection and exclusion criteria, and choice of appropriate databases for the research area; (3) elaboration of the search strategy based on specific descriptors for each database; (4) searching the databases with validation by another researcher who independently evaluated the information; (5) screening and selection from titles and abstracts and reviewing the results with a second independent researcher using the Rayyan tool (https://www.rayyan.ai/) ^18^; (6) calculation of Cohen’s kappa index of inter-rater agreement; (7) full reading of the selected articles and final selection of the analysis corpus; (8) qualitative analysis of the methodological procedures of the included studied based on the Critical Appraisal Skills Program (CASP) ^19^, (9) coding the results of selected articles using the QDAMiner 9.0 Lite program (https://provalisresearch.com/); (10) description and analysis of the material.

This study was registered on the PROSPERO platform (protocol CRD42023451203). The *Enhancing Transparency in Reporting the Synthesis of Qualitative Research* (ENTREQ) guide was used to report the essential elements that must compose a qualitative evidence synthesis ^20^.

### Research question, eligibility criteria and search strategy

Research rigor was ensured by employing the SPIDER strategy ([S] sample; [PI] phenomenon of interest; [D] study design; [E] evaluation; [R] research type) to review studies with qualitative methods ^21^. Thus, the research question was elaborated accordingly: What is the qualitative evidence available in the literature about the sexuality experiences of old cisgender gay men?

Eligibility criteria were set as follows. Inclusion criteria: (a) primary qualitative studies originally published in English, Portuguese, Spanish, or French; (b) studies consistent with the research question developed using the SPIDER strategy; (c) articles that include the experiences of old gay cisgender men aged 50 or older in the results section as categories or subcategories. Exclusion criteria: (a) quantitative, mixed-methods, secondary, or theoretical reflective studies, and literature reviews; (b) gray literature, such as theses, dissertations, monographs, books, or chapters; (c) letters to the editor, editorials, commentaries, opinion articles, and abstracts; (d) studies that did not include cisgender gay men aged 50 or older as participants; (e) studies published in languages other than Portuguese, English, Spanish, or French; (f) studies that did not address sexuality experiences from the perspective of old cisgender gay men.

Database selection considered their relevance to the knowledge area and the objective of covering national and international research scenarios. Subsequently, appropriate keywords were selected according to the specific descriptors for each database. Search queries utilizing the selected keywords were combined based on the SPIDER search strategy. No date restriction was set. Search used the Boolean operators OR for descriptors of the same acronym and AND between each acronym, as follows: (S1 OR S2 OR Sn...) AND (Pi1 OR Pi2 OR Pin...) AND (D1 OR D2 OR Dn...) AND (E1 OR E2 OR En...) AND (R1 OR R2 OR Rn...). The “advanced search” tool was checked. A list of the selected keywords constructed with the Health Science Descriptors (DeCS/MeSH) terms is available in Supplementary Material (Box S1; https://cadernos.ensp.fiocruz.br/static//arquivo/suppl-e00228324_7513.pdf).

### Paper retrieval and selection

Bibliographic search grounded on the formerly described strategy was conducted by two independent researchers in August 2023 on the CINAHL, LILACS, Oasisbr, APA PsycInfo, PubMed/MEDLINE, SCOPUS and Web of Science databases. A total of 2,382 articles were identified during this step. Article selection was further developed using Rayyan software for systematic review, which supports transparent and reliable article screening, reviewing, and selection. After excluding the duplicates (n = 473), the papers underwent a process of blind review and included or excluded by the researchers according to the eligibility criteria. At the end of this step, the blind function was deactivated on the Rayyan tool, revealing the agreements and divergences between researchers’ reviews.

Quality of the agreement between individual reviews was assessed by calculating Cohen’s kappa index. Its calculated value of 0.8502 points to excellent agreement on the review performed by each researcher. As an outcome, 39 articles met the eligibility criteria. Next, the researchers individually read the articles in full, with the eligibility criteria in mind. After in-depth debating, they excluded nine of them, totaling 30 articles included in the meta-synthesis. The studies and reasons for their exclusion are shown in Supplementary Material (Box S2; https://cadernos.ensp.fiocruz.br/static//arquivo/suppl-e00228324_7513.pdf).

**Figure 1:**
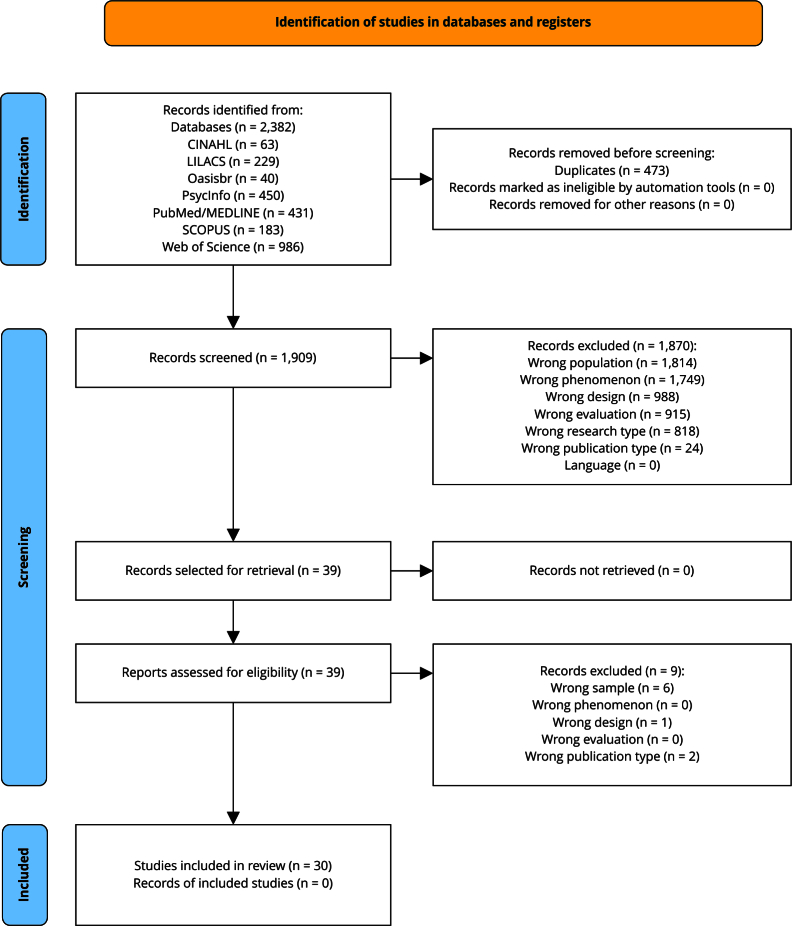
PRISMA (*Preferred Reporting Items for Systematic Reviews and Meta-Analyses*) diagram of the study selection process.

All steps are systematized on Figure 1, a flowchart developed according to the PRISMA (*Preferred Reporting Items for Systematic Reviews and Meta-Analyses*) strategy ^22^.

Details of the 30 studies were systematized and presented according to the following data: study (year), location, objective, design, data collection and analysis, as well as the number and age of participants (Box 1).

**Box 1:** Features of the included studies (n = 30).

STUDY (YEAR)	LOCATION	OBJECTIVE	DESIGN	DATA COLLECTION	DATA ANALYSIS	PARTICIPANTS/AGE RANGE (YEARS)
Waling et al. ^24^ (2023)	Australia	To have a comprehensive understanding of the histories of older lesbians and gay men and how they perceive their lives today	Iridescent life course perspective	Qualitative interviews	Thematic analysis	19/60 and above
Lichelli et al. ^25^ (2023)	United Kingdom	To explore the perceptions of older gay males living with HIV around health and well-being as their experiences as people aging with HIV	Intersectionality	Semi-structured interviews	Thematic analysis	19/50 and above
Willis et al. ^26^ (2022)	United Kingdom	To explore older men’s experiences of loneliness and social participation	Hegemonic masculinities	In-depth semi-structured interviews	Framework approach	21/65-95
Suen ^27^ (2022)	Hong Kong (China)	To understand the dynamics of being an ageing non-heterosexual migrant	Case study	In-depth interview	Case study analysis	1/60 and above
Dhoest ^28^ (2022)	Belgium	To better understand the identifications, experiences, and perspectives of a specific subgroup of the LGBTQ community, GBTQ men	Generational approach	In-depth semi-structured interviews	Thematic analysis	16/60 and above
Fair ^29^ (2021)	United States	To explore the connections between sexuality and spirituality for older LGBTQ adults	Critical theory	In-depth semi-structured interviews	Conceptual content analysis	6/50-80
Marciano et al. ^30^ (2021)	Israel	To examine identity work among older gay men in relation to ICT	Information technology identity	In-depth semi-structured interviews	Reflexive thematic analysis	17/65 and above
Oswald et al. ^31^ (2020)	United States	To explore the social lives of older gay men over 65 years	Constructivist	In-depth semi-structured interviews	Constructivist grounded theory	10/65-77
Löf et al. ^32^ (2020)	Sweden	To investigate how older LGBTQ adults reason about future services when they enter elder care	Status model of recognition	Semi-structured interviews	Thematic analysis	5/70-81
Wilson et al. ^33^ (2018)	Canada	To better understand the experience of older LGBT individuals and to examine their concerns associated with end-of-life	Life course theoretical perspective	Focus groups, semi-structured interviews	Thematic analysis	4/57-78
Tester ^34^ (2018)	United States	To investigate how gay men’s experience varied during the AIDS years and the social factors associated with it	Life course perspective	In-depth interviews	Coding	40/66-88
Pereira et al. ^35^ (2018)	Portugal	To explore the perceptions toward aging among Portuguese gay and bisexual men over 60 years old	Critical gerontology	Structured electronic inquiry	Thematic analysis	15/60 and above
Suen ^36^ (2017)	Hong Kong (China)	To understand how older gay men live with the aging discourse in the gay community	Defensive othering	In-depth life story interviews	Grounded theory, open coding	25/50 and above
Coleman ^37^ (2017)	United States	To gain perspectives about living with HIV from seropositive African-American MSM aged 50 years and older	ND	Focus groups	Content analysis	30/50 and above
Boggs et al. ^38^ (2017)	United States	To assess the perceptions of urban-dwelling LGBTQ older adults on aging in place to inform the development and implementation of an aging in place model	ND	Focus groups, intercept interviews and a Town Hall meeting	Evaluation and process coding	9/50-69
Muraco et al. ^39^ (2016)	United States	To understand which experiences lesbian women and gay men age 50 and older identify as turning points and explore gender differences	Life course theory	In-depth semi-structured interviews	Open coding	20/54-88
Higgins ^40^ (2016)	Ireland	To explore resilience processes among older LGBT adults	Kwon’s resilience framework	In-depth semi-structured interviews	Thematic analysis	22/55-80
Suen ^41^ (2015)	Hong Kong (China)	To explore lived experiences of older single gay men	Critical theory	Interviews	Critical theory	25/50 and above
Neville et al. ^42^ (2015)	New Zealand	To explore the coming out narratives in a group of older gay men	Narrative gerontology	Semi-structured interviews	Narrative analysis	12/65-81
Masten ^43^ (2015)	United States	To explore gay men’s experience of aging with HIV	Life stage theory	In-depth interviews	Grounded theory, open coding	15/54-64
Lyons et al. ^44^ (2015)	Australia	To examine the perceptions about life changes of older gay men	ND	Survey with open-ended questions	Thematic analysis	439/50 and above
Barrett et al. ^45^ (2015)	Australia	To document older LGBT and intersex people’s experiences of discrimination and how these experiences are implicated in higher than average rates of depression and anxiety	ND	In-depth interviews	Thematic analysis	5/65-79
Zhou et al. ^46^ (2014)	China	To explore the range of personal motivators associated with sexual risk behaviors among older male adults	ND	In-depth interviews	Iterative process	14/50 and above
Pilkey ^47^ (2014)	United Kingdom	To explore older gay male experiences of domestic materiality	Queer theory, geographical gerontology	In-depth semi-structured interviews	In-depth analysis	13/50-70 and above
Van Wagenen et al. ^48^ (2013)	United States	To investigate and describe experiences of successful aging in a sample of LGBT older adults	Grounded theory	In-depth semi-structured interviews	Grounded theory, open coding	11/60-80
Rowan et al. ^49^ (2013)	United States	To explore the lived experience of an older gay man living in rural America	Person-environment fit theory	In-depth semi-structured interview, life review	Thematic analysis	1/80
Kushner et al. ^50^ (2013)	New Zealand	To explore the ageing experiences of gay men in New Zealand over the age of 65 years	Critical gerontology	In-depth semi-structured interviews	Thematic analysis	12/65-81
Kong ^51^ (2012)	Hong Kong (China)	To document the uses gay men make of public/private spaces for same-sex intimacy	Post-structuralist power-resistance paradigm	Oral histories, semi-structured interviews, focus groups	Thematic analysis	14/60
Clover ^52^ (2006)	London (United Kingdom)	To explore the experiences of older gay men in relation to health, what gaps existed in health and social care services, and how primary care services could better meet the needs or concerns of older gay men	Conversation with a purpose	In-depth semi-structured interviews	Successive approximation	10/60-70
Chapple et al. ^53^ (1998)	Australia	To explore how elderly gay men attach themselves to gay community in terms of an analysis of class, generation, and the interplay of self-construction and masculinity	Theorized life history	Semi-structured interviews	Thematic analysis	8/41-59

ICT: information and communication technologies; MSM: men who sex with men; ND: non-disclosed.

Source: prepared by the authors.

### Methodological quality of the articles

The methodological characteristics of the selected articles were assessed using the *CASP Qualitative Checklist*, an inventory that guides qualitative evaluation by highlighting content features considered critical for the consistency of qualitative research. Following this approach, two independent reviewers analyzed the included studies evaluating the presentation of the research goals and their importance, the suitability of methodological choices and study design on achieving said goals including data collection and analysis schemes. Ethical procedures were also evaluated ^19^. Next, reviewers produced two lists and compared then, discussing disagreements to reach a consensus. The methodological consistency of all studies was deemed suitable as appropriate qualitative studies. A detailed presentation of this process can be found in Box 2.

**Box 2:** Quality appraisal of included studies according to the Critical Appraisal Skills Program (CASP).

STUDY (YEAR)	1	2	3	4	5	6	7	8	9	10
Waling et al. ^24^ (2023)	Yes	Yes	Yes	Yes	Yes	Can’t tell	Yes	Yes	Yes	Yes
Lichelli et al. ^25^ (2023)	Yes	Yes	Yes	Yes	Yes	Yes	Yes	Yes	Yes	Yes
Willis et al. ^26^ (2022)	Yes	Yes	Yes	Yes	Yes	Can’t tell	Yes	Yes	Yes	Yes
Suen ^27^ (2022)	Yes	Yes	Can’t tell	Yes	Yes	Yes	Yes	Can’t tell	Yes	Yes
Dhoest ^28^ (2022)	Yes	Yes	Yes	Yes	Can’t tell	Yes	Yes	Yes	Yes	Yes
Fair ^29^ (2021)	Yes	Yes	Yes	Yes	Yes	Yes	Yes	Yes	Yes	Yes
Marciano et al. ^30^ (2021)	Yes	Yes	Yes	Yes	Yes	Yes	Yes	Yes	Yes	Yes
Oswald et al. ^31^ (2020)	Yes	Yes	Yes	Yes	Yes	Yes	Can’t tell	Yes	Yes	Yes
Löf et al. ^32^ (2020)	Yes	Yes	Yes	Yes	Yes	Yes	Yes	Yes	Yes	Yes
Wilson et al. ^33^ (2018)	Yes	Yes	Yes	Yes	Yes	Can’t tell	Yes	Yes	Yes	Yes
Tester ^34^ (2018)	Yes	Yes	Yes	Yes	Yes	Yes	Yes	Yes	Yes	Yes
Pereira et al. ^35^ (2018)	Yes	Yes	Yes	Yes	Yes	Can’t tell	Can’t tell	Yes	Yes	Yes
Suen ^36^ (2017)	Yes	Yes	Yes	Yes	Yes	Yes	Yes	Yes	Yes	Yes
Coleman ^37^ (2017)	Yes	Yes	Yes	Can’t tell	Yes	Yes	Yes	Yes	Yes	Yes
Boggs et al. ^38^ (2017)	Yes	Yes	Yes	Yes	Yes	Yes	Yes	Yes	Yes	Yes
Muraco et al. ^39^ (2016)	Yes	Yes	Yes	Yes	Yes	Yes	Yes	Yes	Yes	Yes
Higgins ^40^(2016)	Yes	Yes	Yes	Yes	Yes	Can’t tell	Yes	Yes	Yes	Yes
Suen ^41^ (2015)	No	Yes	Yes	Yes	Yes	Yes	Yes	Yes	Yes	Yes
Neville et al. ^42^ (2015)	Yes	Yes	Yes	Yes	Yes	No	Yes	Yes	Yes	Yes
Masten ^43^ (2015)	Yes	Yes	Yes	Yes	Yes	No	No	Yes	Yes	Yes
Lyons et al. ^44^ (2015)	Yes	Yes	Yes	Yes	Yes	Can’t tell	Yes	Yes	Yes	Yes
Barrett et al. ^45^ (2015)	Yes	Yes	Yes	Yes	Yes	Yes	Yes	Yes	Yes	Yes
Zhou et al. ^46^ (2014)	Yes	Yes	Yes	Yes	Yes	Can’t tell	Yes	Yes	Yes	Yes
Pilkey ^47^ (2014)	Yes	Yes	Yes	Yes	Yes	Yes	Yes	Yes	Yes	Yes
Van Wagenen et al. ^48^ (2013)	Yes	Yes	Yes	Yes	Yes	Yes	Yes	Yes	Yes	Yes
Rowan et al. ^49^ (2013)	Yes	Yes	Yes	Yes	Yes	Yes	Can’t tell	Yes	Yes	Yes
Kushner et al. ^50^ (2013)	Yes	Yes	Yes	Yes	Yes	No	Yes	Yes	Yes	Yes
Kong ^51^ (2012)	Yes	Yes	Yes	Yes	Yes	Yes	Yes	Yes	Yes	Yes
Clover ^52^ (2006)	Yes	Yes	Yes	Yes	Yes	Yes	Yes	Can’t tell	Yes	Yes
Chapple et al. ^53^ (1998)	Yes	Yes	Yes	Yes	Yes	No	No	Yes	Yes	Yes

Source: prepared by the authors.

Note (questions): 1 - Was there a clear statement of the research goals?; 2 - Is a qualitative methodology appropriate?; 3 - Was the research design appropriate to address the research goals?; 4 - Was the recruitment strategy appropriate to the research goals?; 5 - Were the data collected in a way that addressed the research issue?; 6 - Has the relationship between researcher and participants been adequately considered?; 7 - Have ethical issues been considered?; 8 - Was the data analysis sufficiently rigorous?; 9 - Is there a clear statement of findings?; 10 - How valuable is the research?

### Data analysis

Data was investigated by thematic analysis ^23^ following the steps: (1) full reading and line-by-line coding of the selected studies using the QDAMiner Lite software; (2) reviewing and grouping the codes into descriptive themes, similar categories that describes the results of the analyzed studies; (3) development of the analytical themes: new interpretations and constructs based on the original data. Finally, the analysis was discussed and validated with help from the research group in which the researchers participate.

## Results

Although bibliographic research included two databases from Latin America (Oasisbr and LILACS), no studies conducted on this region met the inclusion criteria. We reviewed a total of 30 articles ^24^^,^^25^^,^^26^^,^^27^^,^^28^^,^^29^^,^^30^^,^^31^^,^^32^^,^^33^^,^^34^^,^^35^^,^^36^^,^^37^^,^^38^^,^^39^^,^^40^^,^^41^^,^^42^^,^^43^^,^^44^^,^^45^^,^^46^^,^^47^^,^^48^^,^^49^^,^^50^^,^^51^^,^^52^^,^^53^, with publication dates ranging from 1998 to 2023. Most studies were conducted in the United States (n = 9) and collected data using individual in-depth semi-structured interviews (n = 25) ^24^^,^^25^^,^^26^^,^^27^^,^^28^^,^^29^^,^^30^^,^^31^^,^^32^^,^^33^^,^^36^^,^^39^^,^^40^^,^^41^^,^^42^^,^^43^^,^^45^^,^^46^^,^^47^^,^^48^^,^^49^^,^^50^^,^^51^^,^^52^^,^^53^. Content exploration mainly applied thematic analysis (n = 14) ^24^^,^^25^^,^^28^^,^^30^^,^^32^^,^^33^^,^^35^^,^^40^^,^^44^^,^^45^^,^^49^^,^^50^^,^^51^^,^^53^. The main themes and results of the articles are available in Supplementary Material (Box S3; https://cadernos.ensp.fiocruz.br/static//arquivo/suppl-e00228324_7513.pdf).

Older cisgender gay men belong to the men who have sex with men (MSM) category, which includes bisexual men and other sexual minorities, framing them by their sexual practice. We use “gay men” instead, as it (1) specifies this population and (2) enables an analysis of sexual health beyond sexual activity. Some studies worked with samples composed by other populations besides cisgender gay men, like cisgender women, transgender women, lesbian women, bisexual men, transgender men and heterosexual men ^24^^,^^26^^,^^29^^,^^32^^,^^33^^,^^35^^,^^38^^,^^40^^,^^45^^,^^46^^,^^48^. On these cases, reviewers identified the exact number of cisgender gay men on each study, except for one ^33^. Lower age limit was set at 50 to align with international studies which define this bracket as the start of old age ^25^^,^^36^^,^^37^^,^^38^^,^^41^^,^^44^^,^^47^. One study ^53^ included participants aged 41 to 59 years, but only data from those older than 50 was analyzed. Equal procedure was applied to studies on the broader older LGBTQIAPN+ population, with their information identified in the results section. A total of 849 individuals participated in the 30 studies included in this meta-synthesis. Most studies had a sample size between 10 to 30 participants (n = 19) ^25^^,^^28^^,^^29^^,^^30^^,^^31^^,^^32^^,^^35^^,^^36^^,^^37^^,^^41^^,^^42^^,^^43^^,^^45^^,^^46^^,^^47^^,^^48^^,^^50^^,^^51^^,^^52^.

Free coding of the content extracted generated a total of 81 codes which were further reviewed and refined into 46 codes. After review, they were synthesized into four descriptive themes: (1) Living history: perceptions on the impact of historical and cultural development on experiencing sexuality; (2) Aging as a gay man: understandings on becoming and being a new body and self; (3) Caring for a social network: severing, building and maintaining bonds; (4) Handling health and professional care. The resulting content was then further developed into three analytical themes: (1) (Un)doing gay men through a dehumanizing gaze, (2) Oldness as performance, (3) To care is to care about. These results are detailed in Box 3.

**Box 3:** Codes and themes produced through thematic synthesis.

CODES (LINE-BY-LINE CODING)	DESCRIPTIVE THEMES	ANALYTICAL THEMES
Gay men can’t be old	Living history: perceptions on the impact of historical and cultural development on experiencing sexuality	Analytical theme 1: (Un)doing gay men through a dehumanizing gaze Analytical theme 2: Oldness as performance Analytical theme 3: To care is to care about
The consequences of HIV/AIDS		
The past was worse		
Legislative and police oppression		
Pressure into monogamy		
Heteronormativity		
Homophobia		
Ageism		
Stereotypes for being elderly		
Stereotypes for being gay		
Religious discrimination		
Racial differences		
Coming out is essential for happiness	Aging as a gay man: understandings on becoming and being a new body and self	
Getting old allowed self-acceptance		
The lack of a name for “being gay”		
Territorial impact on experience		
Marriage to women		
Feeling invisible and undesirable		
Being gay can be good		
Being men		
The lack of erotic spaces		
Family is important		
Being old hampers an active sexual life		
Recurrent death of acquaintances		
Elderly’s body is fetishized		
Corporal changes		
I still like my body		
Having sex is important		
Financial changes		
Housing		
Friendships as a source of support	Caring for a social network: severing, building and maintaining bonds	
Isolation is common and harmful		
The lack of a work space is harmful		
Belonging to a community		
LGBTQIA+ community as support		
Positive conjugality		
Negative conjugality		
Being single can be good		
Religion as a place for socializing		
Socializing with the young is hard		
Being cared by a partner	Handling health and professional care	
Self-care		
Fear of seeking care for being gay		
Being neglected for being gay		
Healthcare with hospitality for gays		
Living with HIV is a hardship		

Source: prepared by the authors.

### Descriptive theme 1 - Living history: perceptions on the impact of historical and cultural development on experiencing sexuality

Participants reported that their sexuality was affected by the social and cultural conditions of their youth, contrasting sharply with their old age. The traumatic HIV crisis brought lasting changes, transforming the sexual freedom they experienced at the time into feelings of shame, guilt, and fear ^43^^,^^44^ and resulted in rising homophobia ^44^. Lack of knowledge about the virus contributed to AIDS being labeled as a “gay disease” ^28^, leading to views of being gay as a health hazard ^28^^,^^44^. This stigma prompted many to avoid sex or adapt their sexual practices by learning about safe sex ^28^^,^^34^^,^^37^. Many participants devoted themselves to caring for affected peers ^24^^,^^27^^,^^31^, enduring profound grief as numerous friends died ^24^^,^^31^^,^^34^^,^^43^^,^^44^: “*and then there was a turning point when AIDS came in and everybody died*” ^39^ (p. 129). The crisis was described as a “tragedy”, a “war” or “holocaust” ^28^^,^^31^^,^^43^^,^^44^, leaving survivors with lasting grief and guilt. Fear of further loss made building relationships difficult ^31^^,^^34^^,^^43^^,^^44^. Participants noted that this history shaped their sexuality differently from younger gay men, making them more serious about safe sex and creating a generational experience gap that complicates intergenerational relations ^28^^,^^44^.

Participants noted that the government and laws at the time were a source of oppression that impacted their current perceptions of sexuality. Many feared imprisonment for being gay, which led them to develop strategies to conceal their sexuality ^44^. Socializing posed risks and took place in hidden venues like public toilets and bars ^31^^,^^41^^,^^42^: “*you had to be extremely careful, because the police used to have people* (...) *going around the toilets arresting people. It was a dreadful time*” ^34^ (p. 354-5). This fostered feelings of fear, terror, and a sense of a “lost gay youth” ^24^^,^^31^^,^^44^. Some men still fell unsupported by legislation and the government ^24^^,^^27^^,^^37^^,^^38^^,^^44^.

Beyond prohibitions and punishments, they also expressed feeling oppressed by the obligations and duties assigned to them by a heteronormative way of life. They were expected to work, marry a woman, have children and attend church. This led many of them to live a double life and have secret relationships with men simultaneously to their marriages ^42^^,^^51^. Those who attended Catholic churches commonly experienced a growing sense of shame after repeatedly being told that their sexuality was a sin ^28^^,^^29^^,^^38^^,^^40^^,^^42^^,^^44^^,^^53^. This feeling led these men to fear being found out and labeled as “sick”, “deviant” or “perverted”, to feel apprehensive for whom they were and to hope that they would be able to live a heteronormative life one day ^31^^,^^37^^,^^41^^,^^44^^,^^45^. Nevertheless, worrying about being single was not a concern solely to those who aspired to emulate a “straight” way of life, with participants reporting also feeling pressured to be in a relationship with other men. They considered monogamy as a primary site for intimacy, and linked being single with loneliness ^26^^,^^28^^,^^37^^,^^41^^,^^44^^,^^48^.

When talking about their present lives, most of them perceive a gradual improvement on the social acceptance of homosexuality. They emphasize how important it is for them that the gay youth recognize this new social setting as a results achieved by the political resistance of older cisgender gay men and women in the past ^44^. On their intimate lives, they reported that their families and friends begun presenting a more positive attitude towards gay men, which led many to disclose their sexuality ^35^^,^^44^. They also fell that this freedom is being expressed more broadly, meaning that gay men are more confident to disclose their sexuality on the streets and to openly talk about it on the mainstream media ^28^^,^^35^^,^^41^^,^^44^. However, many believe that further development has to be achieved ^44^. In their perspective, this new found freedom was restricted to the younger generations since older gay men lost their whole social network during the AIDS crisis ^44^. Many reiterated that had they been afforded the same freedom, they would have found a partner to share their lives with during their youth ^41^. This leaves them feeling jealous of the freedom gay youth seem able to enjoy, especially when it comes to expressing their sexuality in ways that goes beyond being gay or cisgender ^28^. Some participants also shared that having to think about being straight or gay was hard, but it feels easier than having to reflect about this new range of possibilities ^28^. Some believed that rejection after coming out in their present seems harsher than in the past, considering that everyone knows what being gay means ^28^. Being unable to benefit from this new social setting is intensified because these men believe that the younger generations usually “turn their back” on them ^35^.

This feeling of being ignored by the gay youth pointed to greater issues. Participants attributed their rejection by younger generations to ageism ^26^^,^^31^^,^^43^^,^^44^^,^^51^^,^^52^, described as a “youth-oriented” culture ^51^. In their perspective, those who “look their age” are ignored and feel invisible among gay men ^36^. Namely, they associate being mistreated to how much “oldness” is shown on their bodies to others ^36^^,^^37^^,^^43^^,^^44^. This led some participants to feel suspicious when approached by younger men, as if these were looking for someone to fill a “paternal” role in their lives instead of actually desiring them ^31^. Other participants declared themselves ageists for not desiring men who “look old” ^36^.

Importantly, the perception these men have about cultural and social aspects are deeply intertwined with where they lived in. To an extent that some even expressed radical shifts on their perspectives when moving from one place to another ^24^^,^^27^^,^^28^^,^^40^^,^^49^.

### Descriptive theme 2 - Aging as a gay man: understandings on becoming and being a new body and self

This theme presents the personal development of these gay men as they aged. In youth, many struggled to understand and manage their desires for other men. Participants noted a lack of vocabulary to describe the sexual practices and desires they were discovering ^24^^,^^28^^,^^30^^,^^42^^,^^51^ which made identifying as gay confusing: “*I was interested in men but didn’t realize it or know anything about it. Who’s gay? What’s gay? It was underdeveloped*” ^30^ (p. 28). Some, however, experienced freedom while not labeling themselves, avoiding concerns about being straight, gay, or bisexual ^40^. They attributed their ignorance of sexuality terminology to the absence of internet and media discussions on the topic ^24^^,^^30^. Many identified as gay only in their late 30s ^30^. Bullying was a common way to force awareness about their sexuality: “*When I was 12, this boy in my class said: ‘Hey, that guy’s a faggot’. I didn’t know what a faggot was, but then I started thinking and said: ‘Yes, maybe I am a faggot’*” ^28^ (p. 4).

During self-discovery, many married women early in adulthood due to heteronormative and monogamist social pressure ^24^^,^^28^^,^^30^^,^^35^^,^^39^^,^^40^^,^^41^^,^^43^^,^^44^^,^^46^^,^^52^^,^^53^. For some, intimacy with their wives was difficult and lead them to avoid sex through excuses like work fatigue, late hours, or keeping their children in the same room ^51^. Others did not face such issues and engaged sexually with both their wives and other men ^42^. Still, participants described marriage to women as distressing, calling it a “punishment”, “heavy burden”, or a “growing cancer” ^30^^,^^40^. Divorce brought feelings of relief and happiness ^38^^,^^40^^,^^45^^,^^49^, but many cherished having children from these marriages ^40^^,^^44^^,^^51^.

Such value placed on having children highlights the importance of family in their lives. Acceptance and support from relatives were deemed fundamental ^24^^,^^35^^,^^40^^,^^44^^,^^45^^,^^47^^,^^53^, underscoring the critical role of coming out. Concealing their sexuality was seen as a heavy burden, a source of frustration and sadness ^27^^,^^30^^,^^40^^,^^50^ linked to difficulties in socializing and feeling isolated ^43^^,^^48^. Coming out was described as a turning point, bringing feelings of liberation, happiness, and emotional relief ^39^^,^^42^^,^^44^^,^^46^. But many feared the potential consequences, considering the risks too great ^24^^,^^28^^,^^30^^,^^35^^,^^43^^,^^45^^,^^48^^,^^52^. For some, coming out led to losing their entire support network, particularly those with Christian backgrounds ^44^.

In old age, participants noted significant bodily changes. Some still “felt young”, attributing aging more to mindset than physical shifts ^36^^,^^52^. Terms like “slowing down”, “a downhill slope”, and “deteriorating” were used to describe their perceptions of physical aging ^52^. Chronic illnesses (polio, arthritis, heart disease) and reduced hearing, vision, or physical responsiveness made daily tasks harder and time consuming ^31^^,^^43^^,^^48^^,^^52^. Features like body odor, gray hair, skin pigmentation, and weight gain were associated with an “old body” ^46^. Many reported less sexual activity ^43^, often due to erectile dysfunction or libido loss linked to medication ^44^.

One crucial factor for participants was dealing with hardships while finding sexual partners, which greatly impacted their well-being, self-esteem, and happiness. Loneliness was a commonly reported issue, especially for those without children ^41^^,^^52^. In their youth, sexual encounters helped build support networks: “*your entry is sex, but it exposed you to so much more if you knew to take advantage of it*” ^31^ (p. 231-2). Currently, those engaging in the “gay scene” (clubs, bars, saunas, beats) fell invisible and rejected ^26^^,^^36^^,^^37^^,^^43^^,^^44^^,^^50^^,^^51^^,^^52^. One participant described feeling “*...unattractive, unfit, which in the gay world means you are dead*” ^43^ (p. 21). Many believe youthfulness dominate the gay community, limiting their inclusion: “*the gay scene is so youth-oriented that you can’t get into it... what space is left for you?*” ^51^ (p. 908). Some resorted to transactional sexual relationships ^36^^,^^41^ or abandoned the pursuit of partners despite still desiring companionship ^35^. This exclusion led many to feel that their elder status conflicted with their gay identities: “*if to be gay you have to be actively gay, I’m not gay anymore*” ^43^ (p. 328). Some participants perceived these challenges as territorial, noting that depending on the country or urbanization level they found partners more easily ^27^^,^^49^.

Using the internet for sex and intimacy offered an alternative ^41^^,^^44^^,^^46^, but not all participants embraced technology ^30^. Some struggled to use it and felt younger generations lacked patience to teach them ^48^^,^^49^. Others feared being discovered by their families when seeking sex online ^30^. Preference for phone or in-person communication are common, with some feeling online interactions lack genuine connection ^44^^,^^49^. Those seeking serious relationships perceive dating apps as focused only on casual sex ^28^.

Overall, the sum of bodily changes and hardships faced in their sexual lives contribute to emerging processes of grief: “*I actually mourned the loss of a part of myself...*” ^31^ (p. 233). Some coped positively, finding value in interests beyond sex ^31^. Those who managed to overcome grief described becoming less worried and experiencing a growing sense of contentment with life ^43^^,^^52^. Some associated aging to feeling greater openness and acceptance about their own sexuality, which led to deeper, more meaningful sexual and emotional connection with other men ^24^^,^^43^^,^^40^^,^^44^^,^^53^: “*It’s so much easier to open about what you do and who you do it with*” ^44^ (p. 2243).

### Descriptive theme 3 - Caring for a social network: severing, building and maintaining bonds

Many participants had few peers, as the AIDS crisis took most of their friends ^24^^,^^31^^,^^39^^,^^43^^,^^44^. Some linked their loneliness to being prohibited from socializing as gay men during this time ^41^. In old age, the death of relatives, friends and partners became a common experience ^39^^,^^45^^,^^49^: “*that’s the trouble when you get old... All your friends start dying...*” ^49^ (p. 195). These factors led to isolation and hindered sociability ^44^. Participants preferred staying home, as it felt safer than a world where they felt secluded from ^37^^,^^43^^,^^45^^,^^51^. Black and HIV-positive gay men, in particular, felt especially vulnerable: “*you feel like you are out there on some island by yourself, and no one cares*” ^37^ (p. 489).

Working and retirement had crucial impacts on their social networks. They valued careers that allowed them to disclose and talk about their sexuality and feel accepted for who they were ^39^^,^^45^^,^^49^. Being able to contribute to the LGBTQIAPN+ community while doing community work was associated with a sense of fulfillment ^31^. Overall, participants described hardships on building careers and feel that opportunities were “snatched out” of their lives. Retirement made things more difficult, as it usually meant a decrease in income and being away from workplaces, were they felt seen, appreciated and could socialize ^25^^,^^31^^,^^39^^,^^43^. Additionally, despite the forementioned difficulties when engaging with religion, frequenting places to practice their faith was considered an important site for socialization and feeling included in something bigger ^29^^,^^43^^,^^44^.

Participants felt divided when talking about the LGBTQIAPN+ community and venues catered towards it as sites for improving their social lives. For some, such spaces were considered to be crucial and the only space possible for queerness ^26^^,^^28^^,^^32^^,^^37^^,^^41^^,^^44^^,^^48^, somewhere they could feel less invisible ^31^^,^^44^. One participant stated that these places “*provide friendship... you know it’s doing us a service, that’s something we require, you can’t just sort of isolate yourself*” ^45^ (p. 136-7). Nonetheless, those who still feared coming out felt unable to seize these spaces in the same manner ^48^. There were also participants who believed that the LGBTQIAPN+ community did not exist anymore, especially when compared with what they believed a community to be during their youth ^26^^,^^44^.

Another impactful factor on their social lives was conjugality. While many regretted spending several years of their lives married to women, the children they had through these relationships provided a feeling of security and well-being. After divorce, some participants found and married other men and describe lasting relationships as an important source of emotional security ^35^^,^^39^^,^^44^^,^^49^. One men, for example, reported that his partner helped him to change his belief that homosexuality was “a sickness” ^44^. Being able to legally marry their partners and having their relationship recognized and accepted by their families was considered extremely important for their happiness ^35^^,^^45^^,^^47^. However, this led some men to center all their social needs on their partners which resulted in toxic situations. Some felt that they had to repress physical and emotional distress to be able to remain engaged ^43^. Others did not feel loved but did not “have the nerve” to leave their marriages ^48^. Some also disclosed difficulties handling aggressive partners or discovering that their partners were HIV-positive ^27^. As their partners were their only source of support, their death became a sum of grief and feeling isolated from the world ^52^. Notably, however, the death of a partner also led some men to take action and expand their social network ^26^. For those who were in relationships were they perceived an “age gap”, feeling that dying before their partners was inevitable was an issue for the relationship: “*it’s inevitable that I will die before him. I am going to die 20 years before him. It’s a common thing in all these ‘ill-matched’ relationships*” ^27^ (p. 4). This last factor is part of a general difficulty these men face when developing intergenerational social relationships.

Despite all the pressure to be into monogamous relationship, some men described being able to thrive in singlehood. For them, being single meant feeling free, independent and “being your own boss” ^26^^,^^41^^,^^44^.

### Descriptive theme 4 - Handling health and professional care

Self-care was linked to nutrition, meditation, quitting drugs and alcohol, exercising, and psychological treatments ^28^^,^^40^^,^^43^. It was primarily motivated by fears of illness, disease, and physical decline ^24^^,^^25^^,^^31^. Some also engaged in self-care to stay “in good shape” and increase their chances of attracting sexual partners, especially younger ones ^36^. The fear of becoming dependent and lacking someone to care for them was widespread and those who lost partners or expected to outlive their current ones faced uncertain futures ^27^^,^^31^^,^^45^^,^^48^. Lack of children was also a common concern, highlighting the connection between parenthood and health care in participants’ perception ^26^^,^^38^. Participants often felt that society did not care about them which, in some cases, hindered health care seeking.

Participants expressed apprehension when seeking professional health care ^27^^,^^35^^,^^50^^,^^52^, fearing that disclosing their sexuality would lead to poorer treatment. Even those who discussed their sexual lives were hesitant to share details like the number of partners ^52^, which led them to avoid asking questions or addressing concerns about sexual health. Opinions on senior care facilities were mixed. Some believed that LGBTQIAPN+-exclusive facilities would prevent them from pretending to be heterosexual or facing mistreatment ^27^, whereas others viewed them as segregationist and felt older cisgender gay men should receive care in conventional facilities ^35^. HIV-positive participants felt overlooked due to their age, stating that HIV had made their entire lives more challenging ^25^^,^^37^^,^^43^^,^^44^: “*I was* [in my 20s] *when I was diagnosed with HIV, it’s all my life really, it’s almost impossible to separate the two...*” ^25^ (p. 6). They reported receiving less support, fewer tests, and less attention to their specific needs from health care professionals, contributing to a perceived higher rate of HIV infection among them ^37^.

Participants reported frequent discrimination from doctors after disclosing their sexual orientation ^30^^,^^31^^,^^37^^,^^48^^,^^52^^,^^53^. One men, describing a consultation about lumps under his arms, recalled: “*...couldn’t get near me! He couldn’t get near me! The way he was, his body language, he wouldn’t touch me*” ^53^ (p. 46). They also felt verbally and mentally abused. Some had undergone conversion therapy in their youth, which they described as a severe trauma with lasting consequences in their present lives ^30^^,^^45^^,^^48^.

Participants also shared experiences with health care professionals that made them feel supported. Knowing they could come out without negative consequences was important: “*I would like to learn in advance that it was OK to say that I’m gay...*” ^52^ (p. 46). Feeling free to discuss their sexuality and sexual practices without judgment or discrimination was a key factor ^28^^,^^32^^,^^50^^,^^52^. They emphasized the importance of being treated with kindness and respect, which was achieved when professionals showed genuine interest in their care. Health professionals expressing concern for their feelings was perceived as a reaffirming attitude ^28^^,^^35^.

### Analytical theme 1 - (Un)doing gay men through a dehumanizing gaze

This Analytical theme examines Descriptive themes 1 and 2, emphasizing how historical, cultural, and social factors shape older cisgender gay men’s self-perception and health care seeking habits. Grounded on the concept of sexual health ^1^^,^^2^, the studies underscore sexuality and care seeking behavior dynamics as phenomena. As such, they must be studied within their specific temporal and spatial context. They cannot be understood definitively; knowledge must evolve to keep up with their ever-changing, contextual nature.

In this case, the HIV crisis and government oppression profoundly impacted gay men’s lives, leaving existential, psychological, and physical consequences. HIV being framed as a “gay disease” in public discourse ^28^ trivialized their suffering and deaths, hindering their recognition as human beings. Despite forming support and care networks, they struggled to resist internalizing the dehumanizing gaze turned on them.

As discussed in Descriptive theme 2, many participants only gained a clearer understanding of their sexuality during youth through exposure to others and culture ^24^^,^^28^^,^^30^^,^^42^^,^^51^ which coincided with being labeled as sick, criminal, sinful or sick ^28^^,^^29^^,^^38^^,^^40^^,^^42^^,^^44^^,^^53^. Their tendency towards isolation and abandoning the pursuit of happiness in later life may reflect a repetition of such harmful narratives, which prevented them from seeing themselves differently. When staying “in the closet” leads to suffering, it can be interpreted as one’s failure to fully develop and embrace one’s personhood.

The creative potential that said gaze ^54^, this way of perceiving themselves and others, has over the sexuality of these men grew as they got older. While many view the increasing freedom and acceptance enjoyed by gay youth today as positive ^28^^,^^35^^,^^41^^,^^44^, it also emphasizes the contrast with their own youth marked by repression and lost opportunities ^24^^,^^31^^,^^41^^,^^42^^,^^44^. Having been forced to “come out” under life-threatening conditions, they now face old age in which their right to desire and have a fulfilling public sexual life is often denied. If exclusion from society as gay individuals represents being “in the closet”, their sense of alienation from the gay community due to age can be seen as a “second closet”. The way some of these old men described not desiring other men their age, for instance, can suggests that they may be reenacting the violent ways in which their bodies and behaviors are seen under a dehumanizing gaze.

Claiming that participants are “(un)done” by a dehumanizing gaze highlights the conditions required for one to be seen as desirable and human. These evolve within historical, social, and cultural contexts, shaping how older cisgender gay men are perceived. Media - movies or pictures, news and narratives - portraying them as sexually inactive, unattractive, or outdated reinforces these perceptions as natural facts. Resuming the concept of gaze ^54^ to analyze these dynamics allows to critically disassemble these representations which in turn can create paths for these men to perceive themselves under new lights.

### Analytical theme 2 - Oldness as performance

As discussed in Descriptive themes 2 and 4, age as a number does not necessarily defines the way in which oldness is perceived. Some participants asserted that they were not old because they did not “act”, “think” or “feel” old ^36^^,^^52^. And when looking for partners, these men considered not “looking old” as a way to have higher chances at being desired as a gay man ^36^^,^^37^^,^^43^^,^^44^. Which leads to the question: if not age, what then counts as acting, thinking, feeling, and looking old? To affirm that oldness can be expressed as a performative act, following Butler’s ^55^ gender performativity, is to argue that the systematic and repetitive use of certain words and representations produces and attributes meanings to bodies and their behaviors that may seem natural and fixed, but in reality must be constantly maintained through repetitive language and cultural practices.

If producing these meanings is seen as performative, they can be transformed by interruptions in the repetitive narratives that frame them as such ^55^. Actions aimed at changing how society and these men perceive “oldness” can significantly impact their health. As noted in Descriptive theme 2, maintaining an active sexual life and participating in erotic spaces is strongly linked to happiness and well-being. If seen as sexually capable, they may feel more confident in occupying spaces that properly welcome them. Moreover, the feeling of being irrelevant to society led participants to neglect seeking care and self-care. Shifting this perspective may foster their inclusion in social spaces, allowing them and others to value their lives, thus creating conditions that facilitate seeking and receiving care.

### Analytical theme 3 - To care is to care about

This Analytical theme suggests that the visibility provided by daily coexistence could improve the quality and frequency of the health care provided to these men, increasing health professionals’ awareness about their health needs. As discussed in Descriptive theme 3, a growing sense of isolation profoundly impacts older gay men’s well-being ^37^^,^^43^^,^^44^^,^^45^^,^^51^. Excluded from their local communities, work and erotic spaces, their sense of self-esteem diminishes which, in turn, compromises their health care seeking behavior ^26^^,^^36^^,^^37^^,^^41^^,^^43^^,^^44^^,^^50^^,^^51^^,^^52^. As presented in Descriptive theme 1, the HIV crisis deeply affected how older gay men engage in seeking and maintaining relationships. This historical event severely affected their social circles and the grief it caused intensified their withdrawal from society, as they feared experiencing similar losses again ^31^^,^^34^^,^^43^^,^^44^.

The hardships faced during this period highlight how effective care strategies can be developed even by those without health care training. Many gay men undertook the responsibility of caring for their peers, who were neglected by professional care ^24^^,^^27^^,^^31^. Amid social chaos, prejudice, and a lack of knowledge or technology to manage HIV, they supported and nurtured each other, valuing their lives until the end ^24^^,^^27^^,^^31^. Thus, to provide health care in all of its forms one must first care about others. The experienced mistreatment reported in Descriptive theme 4 suggests that professionals are not necessarily lacking material resources, but rather failing at the relational aspects of care such as actively listening, being hospitable and creating a welcoming environment for their sexuality. Given how the HIV crisis revealed that emotional engagement is a crucial aspect of care ^24^^,^^27^^,^^31^, older cisgender gay men could potentially receive better care if health professionals developed familiarity with them through everyday interactions. Social participation may help society care for these men, in turn motivating them to seek health care.

## Discussion

Studies on sexual health, rights and justice connect LGBTQIAPN+ movements and political demands to collective health ^1^^,^^2^^,^^4^^,^^56^. Both interdisciplinary fields address social themes previously reduced to biological logic by medical sciences ^1^^,^^2^^,^^4^^,^^56^, highlighting how historical, cultural and social factors shape life and health conditions. Research following these principles can support public health policy development, reinforcing collective health and sexual justice’s shared commitment to improving minorities’ life conditions and health care ^1^^,^^2^^,^^4^^,^^56^.

Understanding the sexual health of older cisgender gay men may benefit from studies on other major populations they’re part of. Studies on men’s sexual health call attention to STIs and erectile dysfunction ^15^ while their presence in health care settings is often overlooked ^57^. Prostate health is particularly critical, as qualitative evidence ^58^^,^^59^ shows men avoid exams due to masculinity views, emotional support deficits, and insecurities about treatment-induced side effects like incontinence and erectile dysfunction.

Gay men highlight “hypervisibility” in their sexual health care, causing needs beyond STI to be overlooked by professionals ^8^. LGBTQIAPN+ sexual health research also emphasizes the need to study subpopulation traits like age or race to improve care strategies for the broader queer community ^14^.

Knowledge on the sexual health of older adults shows that although technological and technical advancements have improved sexual safety and erectile dysfunction among older cisgender men ^60^, professionals often perceive older men as sexually inactive, limiting access to preexposure chemoprophylaxis (PrEP) for HIV prevention ^61^, STI testing, early diagnosis and treatment and a lack of technical knowledge on how to properly provide support and attention to their sexual needs ^12^^,^^37^.

This systematic review and meta-synthesis provide an in-depth understanding of the qualitative evidence on the sexuality experienced by older cisgender gay men. Discussing literature on cisgender men, older adults, and LGBTQIAPN+ offers insights for improving health services, policies, and research. Descriptive themes 2 and 4 shows that how these men are perceived - based on their gender, age and sexuality - impacts their treatment by health providers and their health-seeking behavior ^30^^,^^31^^,^^37^^,^^48^^,^^52^^,^^53^. These findings corroborate knowledge on older adults sexual health, as older cisgender gay men reported a lack of professional support for STI testing ^37^. Notably, their HIV risk is 26 times higher than that of the general population ^62^. These themes highlight their apprehension about seeking care and disclosing sexuality, often justified by past discrimination from health professionals. In practice, this led caregivers to withhold physical examinations and information for these men’s health needs ^53^. Hence, professionals must take a proactive stance, investigating each case with questions to further develop patients’ original demands. This must include health resources and trustworthy care settings where older cisgender gay men feel safe disclosing their intimacy, symptoms, needs, doubts and insecurities. Research with this group can also benefit from these principles.

Descriptive themes 1 and 3 provide insight on the effects past experiences have on their sexuality, social lives, needs and care seeking habits. While research shows that men avoid health services, especially prostate care, due to gender-related issues ^58^^,^^62^ our findings point out that older cisgender gay men avoid socializing and health providers due to fear of discrimination ^30^^,^^31^^,^^37^^,^^48^^,^^52^^,^^53^. The importance these men attribute to their appearance and sexual performance hinders their social lives. Staying secluded in erotic spaces impacts their self-esteem further aggravating isolation ^26^^,^^36^^,^^37^^,^^43^^,^^44^^,^^50^^,^^51^^,^^52^. Professional inquiries can benefit from checking how these men feel about their past and other significant events they believe carers should know about based on an open-ended approach. Importantly, the LGBTQIAPN+ population has been heavily impacted by punitive laws and oppressive government action which criminalizes their sexuality ^24^^,^^27^^,^^31^^,^^37^^,^^38^^,^^41^^,^^42^^,^^44^^,^^62^. Dismantling these laws is linked to health integrity. Developing professional education, public policies and legal support for this population must be accompanied by political resistance advocating for gay civil rights and the depathologization of their sexuality. In short, educational programs for health professionals and research must include information on the history of epidemiological, political, and cultural history affecting the generations they are investigating or caring for.

When caring for or researching about future generations of older cisgender gay men, professionals and researchers should consider the impact of COVID-19 and mpox ^63^. Our findings portrayed abstinence from drug use as a form of self-care ^28^^,^^40^^,^^43^; however, recent studies show that the combination of sexual activity and drug use (chemsex) and transactional relationships are rising globally ^64^^,^^65^. Further investigations on this topic among older cisgender gay men could benefit future actions on sexual health. Advancements in science on these subjects may benefit from reviewing the impact of policies, laws, cultural, and epidemiological factors on their lives.

Brazil has seen important advancements in public policies. Despite no policy specifically targeting the sexual health of older cisgender gay men, existing laws promote their civil rights by safeguarding access to health care to the LGBTQIAPN+ population ^66^^,^^67^^,^^68^^,^^69^. Laws for the general older population affirms that they must receive care tailored to their life stories and contextual conditions ^66^. Men’s health is also promoted by law, but the policy does not address age-related specifics ^69^. The policy promoting LGBTQIAPN+ health mentions that both young and old must receive appropriate care, but it lacks further development ^68^. Consequently, future policies could benefit from the present findings to guide their development and contemplate older cisgender gay men.

Effective prevention and care strategies can extend beyond clinical settings. One key finding of this study is how critical socialization and life stories are to developing health needs for this population, especially family support and acceptance ^24^^,^^35^^,^^40^^,^^44^^,^^45^^,^^47^^,^^53^. To make these life stories known, policies could promote community-based interventions to encourage socialization and strengthen their bonds with relatives and health services. This would enable them to be heard and seen by caregivers, researchers, and the general population. Other LGBTQIAPN+ groups could also benefit from this since psychological distress tends to isolate them ^70^.

The rigorous research procedure described here has limitations. Articles from Latin America were retrieved but failed to meet the inclusion criteria after full reading. Additionally, only one study specifically targeted non-white older cisgender gay men ^37^. Only one study addressed spirituality ^29^, a crucial part of these men histories. Research in these areas may enhance the knowledge on the sexual health of this population. These findings indicate that this field could benefit from Brazilian research, non-white gay men’s experiences, and the relationship between this population’s sexuality and spirituality.

## Conclusion

Our findings can be used to develop interventions to ensure sexual justice by promoting better policies, providing training to health professionals, and raising awareness to the existence of older cisgender gay men in daily life. This study contributes rich and robust descriptions that lay the foundation for planning actions incorporating network-based approaches aligned with public policies and protection, support, and care programs for this population.

Older cisgender gay men are a particularly vulnerable population that requires careful interdisciplinary study. Our data suggests that several factors impacted their life stories, leading them to social isolation and causing psychological vulnerabilities ^70^. Cultural, political, and epidemiological factors - the HIV crisis, the criminalization of their sexuality ^24^^,^^31^^,^^44^ and bad media representation ^28^^,^^44^ - impacted their lives, producing lasting effects on their self-esteem, self-care and care seeking habits ^12^^,^^15^^,^^19^^,^^24^^,^^26^^,^^27^^,^^28^^,^^29^^,^^31^^,^^34^^,^^35^^,^^36^^,^^37^^,^^38^^,^^40^^,^^41^^,^^42^^,^^43^^,^^44^^,^^45^^,^^48^^,^^49^^,^^51^^,^^52^^,^^53^. These men faced difficulties building and maintaining social bonds. Deaths of friends and acquaintances, be during the HIV crisis or as they got older, left them feeling apprehensive of socializing ^24^^,^^25^^,^^26^^,^^27^^,^^28^^,^^29^^,^^31^^,^^32^^,^^35^^,^^37^^,^^39^^,^^41^^,^^43^^,^^44^^,^^45^^,^^47^^,^^48^^,^^49^^,^^52^. Old age also brought changes to their bodies, appearance and sexual capabilities, the derision of which provoked seclusion from erotic spaces ^24^^,^^26^^,^^27^^,^^28^^,^^30^^,^^31^^,^^35^^,^^36^^,^^37^^,^^39^^,^^40^^,^^41^^,^^42^^,^^43^^,^^44^^,^^45^^,^^46^^,^^47^^,^^48^^,^^49^^,^^50^^,^^51^^,^^52^^,^^53^. Older gay men tend to avoid socialization and care services due to past discriminatory experiences related to their sexuality, also expressed by their fear of depending on others ^24^^,^^25^^,^^26^^,^^27^^,^^28^^,^^30^^,^^31^^,^^32^^,^^35^^,^^36^^,^^37^^,^^38^^,^^40^^,^^43^^,^^44^^,^^45^^,^^48^^,^^50^^,^^52^^,^^53^.

Proper sexual justice can only be achieved through political resistance. Health professionals and researchers play key roles in promoting the well-being of this group, which can be further advanced by a call for action for sexual justice. This entails caring for older cisgender gay men in and beyond traditional care and research settings. Scientific inquiries and care geared toward this population need to be allied to political actions to ensure that the citizenship of gay men is properly recognized by the law.
