# Supplementary material for: Experiences of sexuality of older cisgender gay men: a systematic review and meta-synthesis of qualitative studies
Source: Cad Saude Publica. 2025 Jul 25;41(7):e00228324. doi: 10.1590/0102-311XEN228324 (PMC12334169; doi:10.1590/0102-311XEN228324)
Supplement: Supplementary file 1 [file 1678-4464-csp-41-07-EN228324-s.pdf]

SUPPLEMENTARY MATERIAL

Box S1 Elected keywords for the SPIDER search strategy on the PubMed/MEDLINE database.

**S**<br>*Sample*	Men OR Male AND Sexual and Gender Minorities OR Sexual Minorities OR Minorities, Sexual OR Minority, Sexual OR Sexual Minority OR Homosexuals OR Homosexual OR Men Who Have Sex with Men OR Gays OR Gay AND Aged OR Elderly OR Aged, 80 and over OR Oldest Old OR Octogenarians OR Octogenarian OR Centenarians OR Nonagenarians OR Nonagenarian
**Pi**<br>*Phenomenon of Interest*	Sexuality OR Homosexuality, Male OR Male Homosexuality OR Homosexuality, Ego-Dystonic OR Ego-Dystonic Homosexuality OR Homosexuality, Ego Dystonic OR Sexual Health OR Behavior, Sexual OR Sexual Activities OR Sexual Activity OR Activities, Sexual OR Activity, Sexual OR Sex Behavior OR Behavior, Sex OR Sexual Orientation
**D**<br>*Design*	Focus Groups OR Focus Group OR Group, Focus OR Groups, Focus OR Medical Anthropology OR Anthropology, Medical OR Grounded Theory OR Theory, Grounded OR Culture OR Thematic synthesis OR Hermeneutics OR Hermeneutic OR Ethnographic OR Ethnographic Research OR Phenomenology OR Phenomenological Research OR Narrative OR Interviews as Topic OR Interviewers OR Interviewer OR Interviewees OR Group Interviews OR Group Interview OR Interview, Group OR Interviews, Group OR In-depth Interview OR Qualitative Interview OR Content Analysis OR Semantic Analysis
**E**<br>*Evaluation*	Experience OR Experiences OR Sense OR Senses OR Meaning OR Meanings OR Life Change Events OR Events, Life Change OR Life Change Event OR Life Experiences OR Experience, Life OR Experiences, Life OR Life Experience OR Perspective OR Perspectives OR Subjectivities
**R**<br>*Research type*	Qualitative Research OR Research, Qualitative OR Qualitative studies OR Qualitative OR Empirical Research OR Research, Empirical

Source: prepared by the authors.

Box S2 References that were excluded after their full text was reviewed and the reasons for exclusion.

**EXCLUDED REFERENCES**	**REASONS**
Asencio M, Blank T, Descartes L, Crawford A. The prospect of prostate cancer: A challenge for gay men's sexualities as they age. Sex Res Soc Policy. 2009; 6:38–51.	This study aimed to explore the knowledge of gay and bisexual men about the potential sexual consequences associated with prostate cancer and treatments. However, the quotes presented on the results section do not specify if the men are bisexual or gay, thus constituting wrong sample.
Humble ÁM. Moving from ambivalence to certainty: older same-sex couples marry in Canada. Can J Aging. 2013; 32:131–44.	This study aimed to explore the transition to marriage for mid- to later-life same-sex couples. The results do not specify the age or sexuality of the participants' quotes, thus, constituting wrong sample.
McGovern J, Brown D, Gasparro V. Lessons Learned from an LGBTQ Senior Center: A Bronx Tale. J Gerontol Soc Work. 2016; 59:496–511.	This study aimed to explore the impact of an LGBTQ senior center on the lives of the center members'. The sample was composed of LGBTQ individuals and the results sections did not present the participants sexuality, thus, configuring wrong sample.
Træen B, Carvalheira A, Kvalem IL, Štulhofer A, Janssen E, Graham CA, et al. Sexuality in Older Adults (65+)—An Overview of The Recent Literature, Part 2: Body Image and Sexual Satisfaction. Int J Sex Health 2017; 29:11–21.	This study aimed to provide an overview of the literature published between 2005 and 2015 regarding sexual satisfaction and body image in older adults. However, the results were based on a literature review, with no primary qualitative data collected or analyzed. Thus, constituting wrong research type.
Wallach I, Brotman S. The intimate lives of older adults living with HIV: A qualitative study of the challenges associated with the intersection of HIV and ageing. Ageing & Society 2018; 38:2490–518.	This study analyzed the intimate lives of people living with HIV. The sample was composed of heterosexual, homosexual and bisexual men and women. However, the quotes presented on the article do not specify if the men are gay or bisexual, presenting them as 'MSM'. Thus, constituting wrong population.
Gomes HV, Araújo LF, Salgado AGAT, Jesus LA, Fonseca LK da S, Alves MES. Envelhecimento de homens gays brasileiros: Representações Sociais acerca da velhice LGBT. Psychol 2020; 63:45–64.	This study aimed to analyze the social representations of Brazilian elderly gay men about aging, homosexuality and homophobia. However, the age of the participants ranged from 18 to 44, thus, configuring wrong sample.
Santos JVDO, Araújo LF. Aging and internalized homophobia among Brazilian gay elderly: a study of social representations. Arq Bras Psicol 2020 Apr; 72:93–104.	This study aimed to investigate the social representations of aging and internalized homophobia among Brazilian elderly gay men. However, the results were composed of individual words that were collected through the method of word association without proper discrimination of which participants provided each reply. Furthermore, the results were analyzed quantitatively, constituting wrong design.
Hurd L, Mahal R. "I'm Pleased with My Body": Older Men's Perceptions and Experiences of Their Aging Bodies. Men Masc 2021; 24:228–44.	This study investigated men's perception of their aging bodies. It had a mixed sample composed of heterosexual, gay and bisexual men. The quotes presented do not specify the participant's sexuality, thus, constituting wrong sample.
Masten J. Aging with HIV/AIDS: The experience of gay men in late middle age [doctoral dissertation]. [New York]: New York University; 2006. 342 p.	This study aimed to investigate the challenges associated with the aging of self-identified gay men. It was published as a doctoral dissertation, thus constituting gray literature.

Source: prepared by the authors.

Box S3 Main themes and results of the included studies (n = 30).

AUTHOR, YEAR	MAIN THEMES	RESULTS
Waling et al. [24], 2023	1. Coming out and concealment, 2. Relationship breakdowns with family, 3. HIV/AIDS, 4. Experiences of discrimination, 5. Confidence and self-esteem, 6. Contentment.	The findings affirm that experiences of stigma and discrimination associated with the HIV/Crisis heavily impacted the participants of the study. Their family relationships and their sense of confidence and self-esteem were compromised as well. These participants, however, also expressed a growing sense of fulfillment and gratitude towards their present lives.
Lichelli et al. [25], 2023	1. Loneliness, loss and social dislocation, 2. Diverging life-events that trigger loneliness; 3. Variations in visibility and exclusion across social settings	The findings affirm that both straight and gay elderly men experience hardships due to loneliness and social isolation. However, data suggested that elderly gay men are particularly affected due to how they're secluded from venues targeted towards LGBTQIA+ socialization. Both groups expressed intentions of seeking social environments where they were welcomed, especially at local institutions and groups.
Willis et al. [26], 2022	1. Loneliness and social isolation, 2. Diverging life-events that trigger loneliness, 3. Variations in visibility and exclusion across social settings	This research produced inquiry on loneliness, social dislocation and invisibility experiences of elderly men who are single or living alone. The results affirm that gay men's experience of lonely differs from straight men due to the heteronormative social environments they had to deal with during their lives. This deprived them of spaces where they could safely discuss their sexual and intimate lives openly. The outcomes reinforce the importance of LGB groups and social spaces and how complex gay men feel at some gay spaces like bars and clubs. The study indicates that understanding the unique isolation experienced by gay men requires examining their perceptions of masculinity and how they manage their sexual identities.
Suen [27], 2022	1. A sexually repressed UK, 2. Migration as liberation, 3. White privilege as a shield from homophobia and ageism, 4. Being in a relationship that is not legally recognized	The findings affirm that the experience of ageing as gay men is linked to territory and citizenship status. The participant reported shifts on how he was able to express and live his sexual life depending on the city and country he was living in. The study reports that rural areas tend to have harsher discrimination against gay men and living in large cities allows them to access a less oppressive social setting. On this case, the participant moved from the UK to Hong Kong, which allowed for greater sexual expression but also meant not being able to legally marry another man.
Dhoest [28], 2022	1. Then: Formative Experiences, 2. Now: Generational Perspectives	The findings analyzed the experience of sexuality of elderly gay men taking a generational approach. The study affirms that men born between 1945 and 1964 faced hardships during the HIV/Crisis heavily impacted the development of their understanding of their own sexuality. By comparing them to other LGBTQIA+ groups and discussing their differences, it suggests future research is required on how younger generations and LGBTQIA+ women handle and perceive their own sexuality.
Fair [29], 2021	1. The Sexual Act as Spiritual, 2. Authentic LGBTQ Journey as Spiritual, 3. Sexuality and Spirituality as Inseparable, 4. Love and/ or Attraction as Spiritual, 5. Ineffability of the Connection	The findings investigated the connection between sexuality and spirituality for the elderly LGBTQIA+. The study affirms that, even though discrimination hindered how elderly LGBTQIA+ access and develop their spirituality, they still turned to it when interpreting and attributing meaning to their lived sexual experiences. Thus, the development of environments where LGBTQIA+ can practice their faith and share their spiritual views could benefit their experience of sexuality.
Marciano et al. [30], 2021	1. Technology's potential role in the participants' "heterosexual" past, 2. Technology and advanced age: alienation and estrangement	This research analyzed identity work among elderly gay men in relation to Information and Communication Technology. The article affirms that technology use has been an important path for gay men who could not access social and erotic spaces during their lives, providing a way for them to connect with other gay men. However, the participants reported hardships using and understanding new technologies, which hindered this resource as a way for them to connect with others. The study suggests that technology is an important locus for these men to develop a dimension of their sexual identities through socialization.
Oswald et al. [31], 2020	1. Coming of age as a gay man in the 20th century, 2. Dealing with the aging body, 3. Enduring loss and the consequent impact on social life	This study developed a model for complex intimacy of elderly gay men. As such, it intended to describe how they managed their aging bodies and the losses they experienced during the HIV crisis by trying to connect to younger generations of gay men and gay environments. The findings support that culturally sensitive gay-affirming aging services are necessary to support these men, especially considering how they relied on these services to cope and handle their needs.
Löf et al.[32], 2020	1. Openness and recognition, 2. Preferences regarding how to be treated in elder care, 3. LGBTQ housing	This study focused on the perception of older LGBTQIA+ adults of elder care services. The findings affirm that being recognized and accepted for their sexuality is important. Consequently, in their point of view, elder care services should provide services that recognize sexual diversity through environments where sexuality can be disclosed without discrimination.
Wilson et al. [33], 2018	1. Social connections and	This research investigated LGBTQIA+ aging and end-of-life. The findings affirm that

	support, 2. Familial support, 3. Staying out of the closet.	gender identity and sexual orientation are key determinants of health. Participants disclosed fearing that their gender identity and sexuality might affect their end-of-life experiences. Fear of isolation and a need for social support networks were deemed critical to their health by the study.
Tester [34], 2018	1. Entrenched in the epidemic, 2. Insulated from the epidemic, 3. Removed from the epidemic.	The findings of this study affirm that elderly gay men's experience of social and spatial relationship to urban gay communities during the HIV crisis shaped their experiences of personal loss, community loss, personal support, and community support. The results are supported by discussions that intended to develop a deep investigation of how elderly gay men experienced the epidemic.
Pereira et al. [35], 2018	1. Positive perceptions of aging, 2. Negative perceptions of aging, 3. Coping with being a gay/bisexual man and family ties, 4. Professional care, 5. Homophobia / discrimination, 6. Relationships and social support, 7. Intergenerational differences, 8. Mediating role of sexual orientation, 9. Socialpolitical change, 10. Personal characteristics.	This study affirms that elderly gay and bisexual men experience the present differently from younger generations. Although several important advancements have been made in terms of cultural and political changes, these men do not benefit or perceive these transformations as young gay men do. The research points out that this factor is related to their past experiences of discrimination, present experiences of ageism on daily life and hardships when dealing with diminishing familiar and communitarian support.
Suen [36], 2017	1. Internalizing an aging body as unattractive, 2. Idealizing younger gay men's bodies, 3. Aging as attractiveness, 4. Defensive othering	This article affirms that the gay community commonly marginalizes elderly gay men due to their aging bodies. Thus, elderly gay men tend to judge their own bodies and behavior in a harsh manner, compromising their self-esteem and socialization with other gay men. The study also points out that elderly gay men tend to defensively apply these standards of youth as a value to other elderly gay men, while exempting themselves from this judgment.
Coleman [37], 2017	1. Feeling left out, 2. No place to call home, 3. Not a priority, 4. No one to grow old with.	The findings of this study analyzed mental health issues associated with aging and HIV for older African American men who have sex with men (MSM). These men faced exclusion from local communities and isolation from gay social networks. Overall, the combined stigma of being African, gay, old and seropositive signaled that these men feel extremely secluded from society, which contributes to a worsening mental health.
Boggs et al. [38], 2017	1. Barriers to aging in place, 2. Strengths of the LGBTQ aging community, 3. Participants recommendations for aging in place model	This study affirms that the support elderly LGBTQ identify as necessary for aging in place is similar to heterosexual older adults. Health, finances, legal services, home assistance related to functional limitations and transportation were highlighted as important needs. The LGBTQ population faced increased isolation and had feelings of fear when it came to home assistance. As a result, the study recommended the development of community-based services, focused on offering environments where elderly LGBTQ can gather to socialize and have access to information.
Muraco et al.[39], 2016	1. Relationships turning points, 2. Occupational turning points, 3. Military service, 4. Coming out, 5. Death and loss	This study analyzed turning points in the lives of elderly lesbians and gay men. The findings suggest many similarities between the straight population and lesbians and gay men. Family transformations and professional success being recurrent topics. However, the study suggests that these similarities occur in nuanced ways, with gender identity and sexual orientation impacting these differences. When it comes to elderly gay men, the study points out how heavily they were impacted by the HIV crisis and how they struggle to develop families through marriage or having children.
Higgins [40], 2016	1. Making a decision accept oneself and not be defined by the LGBT identity, 2. Acquiring an empowering perspective, 3. Learning to let go and moving on, 4. Leaving oppressive social environments, 5. Experiencing affirming relationships with family and others, 6. Accessing formal support, 7. Maintaining connections with LGBT people, 8. Remaining positive and being thankful for life, 9. Staying active and keeping busy	Through research on resilience processes among the elderly LGBT population, this paper discussed how research usually focuses on the vulnerabilities of this group while neglecting the processes that strengthen their health, socialization and self-esteem. The study argues that this framing composes an expanding research field. Its findings suggest that, despite general adversities this population tends to struggle with, the participants of this research developed courage and strength, a positive sense of self and an optimistic outlook on life.
Suen [41], 2015	1. Couplehood culture in society and the social pressure to couple up, 2. Alternative discourse decentring the conjugal couple, 3. Freedom	This paper investigated the experience of singlehood on elderly gay men. The findings indicate that all participants faced social pressure to be in a relationship. Being single was perceived by others as deviant, which the paper address as elderly gay men being forced into accommodating themselves into heterosexual ways of life. Although some participants reported being single by choice and being happy with this, the study

	and independence, 4. Sexual exploration, 5. Being 'single of no choice'	reinforced that monogamous relationship remain as one of the central pathways for socialization for this population. Thus, when they're deprived of chances to build relationships like these or cannot find other ways to socialize, they commonly struggle with social isolation.
Neville et al. [42], 2015	1. Early gay experiences, 2. Trying not to be gay, 3. Acceptance	By researching coming out narratives of elderly gay men, this paper provided findings on how they experienced and overcame stressful and challenging life events. The paper affirms that participants faced a double jeopardy when it came to discrimination due to being gay and old. It presented how some of these men tried 'not being gay' and 'staying in the closet', resulting in emotional distress and further social isolation.
Masten [43], 2015	1 Physical challenges, 2. A magnitude of loss, 3. Internal changes, 4. Stigma	The findings of this research dealt with aging as a gay man living with HIV. It affirms that the HIV crisis had severe impacts on how these men experience social interactions. After having their social networks decimated during this historical moment, they experienced fear of developing new bonds and survivor's guilt. This had lasting effects on their lives, affecting even their present social interactions, leaving many of them unable to build and maintain a social support network.
Lyons et al. [44], 2015	1. Acceptance and recognition, 2. Acceptance concerns, 3. Experiences of gay community, 4. The role of technology, 5. Loss of community, 6. The social impact of HIV, 7. Experiences of aging as a gay man, 8. Sexual changes, 9. Need for companionship	This study investigated how elderly gay men perceived their lives after completing 20 years of age. The results affirm that positive life changes were reported by participants, pointing out that they felt more accepted by other men and were able to engage sexually and socially with the gay community during their youth. As they aged, however, they felt the gay community was youth-oriented and that ageism secluded them from this environment where they once felt accepted in. Those who had supportive families and friendships were able to cope with this isolation. Overall, all participants reported having to deal with solitude.
Barrett et al. [45], 2015	1. Early relationship with biological family, 2. Intimate relationships, 3. Friendship and the broader community, 4. Ageing, disability and support services	This paper analyzed the impacts of homophobia on the lives of elderly gay men and lesbian women. The outcomes of the research point out that family, friendships and other forms of connecting socially are fundamental to the mental health of both group of participants. The lack of this form of support was usually compensated by romantic relationships, with their partners commonly being the only form of support they had.
Zhou et al. [46], 2014	1. Sexual desire/interest remaining high at an older age, 2. Unfulfilled sexual desires within marriage, 3. Homosexual or bisexual orientation, 4. Peer influence, 5. Need to socialize with others, 6. Personal choice of "hobby"	The results from this study dealt with sexual risk behavior among elderly straight, bisexual and gay men. The findings suggest that, while straight men tend to engage in transactional relationships (paid sex), gay men commonly tended towards casual sex. Sexual risk behaviors were considered higher among gay men and results affirmed that drug use was a resource they applied to find social and sexual connections with other men.
Pilkey [47], 2014	1. Materiality at home, 2. Looking beyond sexuality in representations of family	This research investigated the experiences of engagement with domestic material objects among elderly gay men. The reports affirm that the domestic space was valued by participants and many embellished their houses with objects that cherished and celebrated their sexual orientation. The findings suggest that this is not necessarily consciously made, pointing out that the natural way this engagement occurs can indicate a form of coping and resistance to the discrimination they face in public.
Van Wagenen et al. [48], 2013	1. Traditionally successful: avoidance of problems, 2. Surviving and thriving: successfully coping with problems, 3. Working at it: some coping, and with effort, 4. Ailing: mostly struggling with problems	This study investigated successful aging experiences of elderly LGBT. The findings point that having access to resources to care for their needs was crucial to their health. This included being able to care for their physical health and having an active social life. The findings point out that their social lives, physical and mental health were connected and having to cope with problems in one of these domains commonly affected the others.
Rowan et al. [49], 2013	1. Life satisfaction	This paper investigated the lived experiences of an elderly gay man living alone in rural communities. The article suggests that, while literature points out that living in rural areas can pose challenges like discrimination and isolation, the participant was able to thrive by engaging with the local community. The participant reported not struggling with discrimination during his life. However, researchers pointed out that he commonly used words and expressions that indicated he was not public about his sexual orientation. The findings reinforce the importance of engaging in social activities and being part of communities as a crucial factor for life satisfaction.
Kushner et al. [50], 2013	1. Homophobia, 2. Being with someone, 3. Future care	This study was conducted on the aging experiences of elderly gay men. The results point out that although many participants were able to overcome adversities like discrimination and isolation, many struggled with fear when it came to seeking health care services and thinking about needing a long-term care facility in the future. The findings indicate that this is related to past experiences of discrimination and apprehension about having to 'go back in the closet'. Thus, culturally sensitive care is

		recommended as an outcome for clinical practice.
Kong [51], 2012	1. All in the family, 2. The secret garden, 3. The home of one's own, 4. The new Tongzhi space and homonormativity	The findings from this paper dealt with sexual intimacy in everyday sites of elderly gay men. The results point out that public sites, named 'gardens' by the participants, were a common place for socialization and sexual relationships for gay men in the past. Although homosexuality was considered a crime at the time, participants were more impacted by the local moral culture, and were concerned they would bring shame to their families. As they aged, they reported a rise in government regulation of these public settings and felt they were not able to experience the freedom younger generations had with cultural shifts on homophobia due to being old.
Clover [52], 2006	1. Growing older, 2. Experiences of health and social services, 3. Talking openly about being gay	The findings of this study dealt with the relationship between being an elderly gay man and health and social services. The findings suggest that the overall elderly population does not necessarily receive 'uncaring care', but that minorities are at a greater risk of dealing with poor practice. Although health care services are considered to be critical to the health of elderly gay men, the results reinforce the importance of the wider social context, highlighting that friendships, social and community work were critical to the development of their health needs.
Chapple et al. [53], 1998	1. Generational effects in the formation of gay identity, 2. Compulsory heterosexuality, 3. The investment in masculinity, 4. Masculinity and work, 5. Work and homosexual practice, 6. Marriage and homosexual practice, 7. The reconstruction of identity	This paper discusses the findings from an inquiry on the sexual lives of elderly gay men. The findings argue that this population experiences a particular form of masculinity, deeply linked to gay community, how their bodies are perceived and the social acceptance of homosexuality. Although research pointed out that the HIV crisis impacted their lives negatively, it also connected these men to institutions and gay communities that play a key role in providing information on sexuality and social participation.

Source: prepared by the authors.
